# Use of a post-production fractionation process improves the nutritional value of wheat distillers grains with solubles for young broiler chicks

**DOI:** 10.1186/2049-1891-4-18

**Published:** 2013-04-22

**Authors:** Philip Thacker, Aman Deep, Eduardo Beltranena

**Affiliations:** 1 Department of Animal Science, University of Saskatchewan, 51 Campus Drive, Saskatoon, SK, S7N 5A8, Canada; 2 Alberta Agriculture and Rural Development, 7000 113th Street, Edmonton, AB, T6H 5T6, Canada

**Keywords:** Broilers, Digestibility, Fractionation, Performance, Wheat distillers grains with solubles

## Abstract

**Background:**

Post-production fractionation of wheat distillers grains with solubles (DDGS) increases their crude protein content and reduces their fiber content. This experiment was conducted to determine the effects of fractionation of wheat DDGS on apparent total tract digestibility (ATTD) and performance when fed to broiler chicks (0–21 d).

**Methods:**

A total of 150, day-old, male broiler chicks (Ross-308 line; Lilydale Hatchery, Wynyard, Saskatchewan) weighing an average of 49.6 ± 0.8 g were assigned to one of five dietary treatments in a completely randomized design. The control diet was based on wheat and soybean meal and contained 20% regular wheat DDGS. The experimental diets contained 5, 10, 15 or 20% fractionated wheat DDGS added at the expense of regular wheat DDGS.

**Results:**

The ATTD of dry matter and gross energy were linearly increased (*P* < 0.01) as the level of fractionated wheat DDGS in the diet increased. Nitrogen retention was unaffected by level of fractionated wheat DDGS (*P* > 0.05). Weight gain increased linearly (*P* = 0.05) as the level of fractionated wheat DDGS in the diet increased. Feed intake, feed conversion and mortality were unaffected by level of fractionated wheat DDGS in the diet (*P* > 0.05).

**Conclusions:**

Post-production fractionation of wheat DDGS improves their nutritional value by lowering their fiber content and increasing their content of crude protein and energy. These changes in chemical composition supported increased weight gain of broilers fed wheat DDGS.

## Background

There is increasing interest in producing ethanol from cereal grains for use in motor fuel [1]. Ethanol-blended fuels offer several advantages over regular gasoline including protection from gas line freezing and higher octane ratings [2]. Most importantly, ethanol-blended fuels have the potential to reduce motor vehicle greenhouse gas emissions by as much as 30% [3].

To produce ethanol, grain is milled, mixed with water and cooked [4]. Enzymes (i.e. amylases, proteases and xylanase) are added to the mixture to convert starch to sugar and the sugar is fermented by the addition of yeast [5]. After complete fermentation, the ethanol is removed by distillation and the remaining fermentation residues are dried and used for livestock feed [4].

From an economic standpoint, wheat DDGS are an attractive feedstuff for use in poultry rations. However, the relatively high fiber content of wheat DDGS reduces nutrient digestibility and impairs the growth rate of broilers fed diets containing high levels of wheat DDGS [1]. Fractionation technologies are being developed by ethanol plants in an effort to remove non-fermentable components of the grain and improve ethanol yield [6]. Using fractionation technologies to produce ethanol can increase ethanol yield by approximately 10% due to a higher percentage of starch entering the ethanol fermentation tank [7]. Front-end fractionation technology involves separating the endosperm, germ and bran fractions prior to fermentation. This process eliminates the non-fermentable fractions, which are in the germ and the bran [7]. The nutritional value of fractionated corn DDGS has been tested with broilers [7], swine [8] and feedlot heifers [9].

Recently, techniques have been developed to use post-production sieving techniques to produce fractionated wheat DDGS [10]. By running wheat DDGS through a series of sieves, it is possible to obtain a product with a higher crude protein content and a lower fiber content [11]. The nutritional value of this product has not been widely evaluated with poultry. Therefore, this feeding trial was conducted to determine the effects of feeding graded levels of wheat DDGS fractionated post-production on broiler performance and nutrient digestibility.

## Materials and methods

### Production of fractionated wheat distillers grains with solubles

The process used to produce fractionated wheat DDGS has been described by Zhang et al. [11]. Briefly, fractionated wheat DDGS were produced from regular wheat DDGS by particle size and weight separation using continuous flow, vibratory equipment. Separation by particle size was conduced using a SWECO ZS30 vibro-separator (SWECO Inc., Florence, KY), equipped with three circular sieves including 600 μm, 425 μm and 250 μm at a rate of 24 kg/h. The material that remained suspended over the 600 μm sieve was separated by differential weight using a Westrup LA-K gravity separator with feed vibration of 6.5 (0–10), air supply of 1 (0–10), long side inclination of 2.0 and short side inclination of 2.5 at a rate of 23 kg/h. The chemical composition of the fractionated wheat DDGS and regular wheat DDGS are shown in Table 1.

**Table 1:** **Chemical and amino acid analysis of main ingredients used to determine the nutritive value of regular and fractionated wheat distiller’s dried grains with solubles (DDGS) fed to broiler chickens**^**1**^

	**Wheat**	**Soybean meal**	**Wheat DDGS**	**Fractionated wheat DDGS**
Chemical composition, % as fed				
Moisture	10.81	7.94	9.22	9.06
Crude protein	14.10	45.53	37.51	45.79
Ash	1.68	6.21	5.49	5.19
Ether extract	1.90	1.28	3.99	3.55
Neutral detergent fiber	12.05	12.19	26.76	20.44
Acid detergent fiber	3.66	5.28	12.66	8.17
Calcium	0.05	0.34	0.14	0.13
Total phosphorus	0.37	0.57	0.80	0.70
Phytate phosphorus	0.22	0.44	0.17	0.12
Essential amino acids, % as fed				
Arginine	0.60	3.29	1.50	1.71
Histidine	0.30	1.18	0.69	0.83
Isoleucine	0.49	2.15	1.22	1.47
Leucine	0.90	3.47	2.17	2.65
Lysine	0.36	2.92	0.81	0.88
Methionine and cystine	0.51	1.31	1.16	1.43
Phenylalanine	0.67	2.32	1.51	1.82
Threonine	0.37	1.75	0.99	1.18
Tryptophan	0.15	0.66	0.33	0.39
Valine	0.59	2.30	1.54	1.81

^1^All data are the results of a chemical analysis conducted in duplicate.

### Animal care

The birds used in this study were housed and managed according to the Canadian Council on Animal Care Guidelines [12].

### Broiler performance trial

A total of 150, day-old, male broiler chicks (Ross-308 line; Lilydale Hatchery, Wynyard, Saskatchewan) weighing an average of 49.6 ± 0.8 g were assigned to one of five dietary treatments in a completely randomized design. The control diet was based on wheat and soybean meal and contained 20% regular wheat DDGS. The experimental diets contained 5, 10, 15 or 20% fractionated wheat DDGS added at the expense of regular wheat DDGS (Table 2). The experimental diets were formulated to supply 3,100 kcal/kg ME, 1.15% lysine, 0.90% threonine, as well as 0.92% methionine and cystine. DL-methionine was added to ensure that all diets provided a similar level of all essential amino acids. All diets were supplemented with sufficient vitamins and minerals to meet or exceed the levels recommended by the NRC [13]. The experiment diets were provided in mash form (3 mm screen).

**Table 2:** **Diet composition of experimental diets formulated to determine the effects of feeding regular and fractionated wheat distiller’s dried grains with solubles (DDGS) to broiler chicks**^**1**^

	**Level of fractionated wheat DDGS, %**				
**Ingredient, % as fed**	**0**	**5**	**10**	**15**	**20**
Wheat	48.38	48.96	49.54	50.12	50.70
Soybean meal	20.76	20.24	19.72	19.19	18.67
Regular wheat DDGS	20.00	15.00	10.00	5.00	0.00
Fractionated wheat DDGS	0.00	5.00	10.00	15.00	20.00
Canola oil	6.44	6.38	6.31	6.25	6.19
Vitamin-mineral premix^1^	0.50	0.50	0.50	0.50	0.50
Dicalcium phosphate	1.16	1.15	1.13	1.12	1.10
Limestone	1.54	1.55	1.56	1.56	1.57
Salt	0.50	0.50	0.50	0.50	0.50
Endofeed W^2^	0.03	0.03	0.03	0.03	0.03
Choline chloride	0.08	0.08	0.08	0.08	0.08
Chromic oxide	0.35	0.35	0.35	0.35	0.35
D-L Methionine	0.05	0.05	0.04	0.04	0.03
L-Lysine HCl	0.22	0.23	0.25	0.26	0.27

^1^Supplied per kilogram of diet: 11,000 IU vitamin A, 2,200 IU vitamin D_3_, 30 IU vitamin E (dl-α-tocopheryl acetate), 2.0 mg menadione, 1.5 mg thiamine, 6.0 mg riboflavin, 60 mg niacin, 4 mg pyridoxine, 0.02 mg vitamin B_12_, 10.0 mg pantothenic acid, 6.0 mg folic acid, 0.15 mg biotin, 0.625 mg ethoxyquin, 500 mg CaCO_3_, 80 mg Fe, 80 mg Zn, 80 mg Mn, 10 mg Cu, 0.8 mg I, 0.3 mg Se.

^2^Enzyme product produced from Aspergillus Niger fermentation providing 700 units/g of β-glucanase and 2,250 units/g of xylanase (GNC Bioferm, Saskatoon, SK).

This experiment was conducted in an environmentally controlled broiler facility located on the campus of the University of Saskatchewan (Saskatoon, Saskatchewan). The chicks were housed in raised-floor battery cages (83.8 cm × 45.7 cm × 25.4 cm; Jamesway Manufacturing Co., Ft. Atkinson, WI, USA) with mesh grate floors located above excreta collection trays. There were five birds per pen and six replicate pens per treatment. Feed and water were available ad libitum throughout the 21-day experiment. Broilers were weighed at the start (d 1) and end of the experiment (d 21) as well as at weekly intervals. Weighed amounts of feed were added as required with a single weigh back at the conclusion of the experiment to allow for the calculation of feed consumption and feed conversion on a pen basis. The battery brooder was maintained at a temperature of 35°C for the first week with the temperature gradually reduced to 29°C by the end of the second week. All chicks were provided with 23 h of light and 1 h of dark with an intensity of 10 lux throughout the experimental period.

### Digestibility trial

Chromic oxide (0.35%) was added to all diets as a digestibility marker and was fed throughout the experiment. During the final two days of the experiment (morning and afternoon), clean excreta (free from feathers and feed) were collected from plastic liners placed in the excreta collection trays underneath each pen of birds. The excreta samples from the four collections were pooled and then frozen for storage. Prior to analysis, the samples were dried in a forced air oven at 55°C for 72 h, followed by fine grinding (0.5 mm screen) using a centrifugal mill (Retzsch ZM 100, Retzsch GmbH, Haan Germany). The digestibility coefficients for dry matter and gross energy as well as nitrogen retention were determined using the equations for the indicator method described by Schneider and Flatt [14].

Coefficients for apparent total tract digestibility (ATTD) were calculated based on the following equation:

$$\mathrm{ATTD}=1-\left[\left({\mathrm{Cr}}_{\mathrm{diet}}/{\mathrm{Cr}}_{\mathrm{out}}\right)\times \left({\mathrm{Nut}}_{\mathrm{out}}/{\mathrm{Nut}}_{\mathrm{diet}}\right)\right]$$

where Cr_diet_ was the chromic oxide concentration in the diet; Nut_diet_ was the dietary concentration of the nutrient or dietary component being assessed and Cr_out_ and Nut_out_ were the concentrations of chromic oxide and the nutrient/dietary component in the excreta.

### Chemical analysis

Samples of the ingredients, experimental diets and excreta were analyzed according to the methods of the Association of Official Analytical Chemists [15]. Analyses were conducted for moisture (AOAC method 930.15), crude protein (AOAC method 984.13), ash (AOAC method 942.05), neutral detergent fiber (AOAC method 2002.04) and ether extract (AOAC method 920.39). An adiabatic oxygen bomb calorimeter (Parr; Moline, Illinois) was used to determine gross energy. Chromic oxide was determined by the method of Fenton and Fenton [16].

Calcium and phosphorus were determined using the nitric-perchloric acid digestion method of Zasoski and Burau [17] with calcium determined on an Atomic Absorption Spectrophotometer (Perkin-Elmer Model 4000; Waltham, MA) using AOAC method 968.08 while total phosphorus was determined colorimetrically (Pharmacia LKB Ultrospec III, GE Healthcare, Little Chalfont, UK) using a molybdovanadate reagent (AOAC method 965.17). Phytate was determined following the procedures of Newkirk and Classen [18]. The concentration of phytate bound phosphorus in each ingredient was calculated as 28.2% of phytate [19] and non-phytate phosphorus was calculated as the difference between the concentration of total phosphorus and phytate bound phosphorus.

The amino acid content of the diets and ingredients were determined by High Performance Liquid Chromatography (Hitachi L-8800 Amino Acid Analyzer, Tokyo, Japan). All samples were hydrolyzed for 24 h at 110°C with 6 mol/L HCl prior to analysis. Sulphur-containing amino acids were analyzed after cold formic acid oxidation for 16 h before acid hydrolysis. Tryptophan was determined after alkaline hydrolysis at 120°C for 16 h.

### Statistical analysis

All data were analysed as a one-way ANOVA using the Proc-Mixed program of the Statistical Analysis System Institute [20]. Treatment means were also tested for linear, quadratic and cubic effects of graded levels of fractionated wheat DDGS. Differences were considered to be significant when *P* < 0.05.

## Results and discussion

### Chemical composition

A chemical analysis of the main ingredients used in the present study is shown in Table 1. The chemical analyses for the wheat and soybean meal used in the present experiment are within the range of those previously reported for these ingredients in standard industry sources such as Feedstuffs [21], the Novus Raw Material Compendium [22] as well as the National Research Council’s Feed Composition Tables [13].

In comparison with regular wheat DDGS, fractionated wheat DDGS were substantially higher in crude protein (45.79 vs. 37.51%) and lower in neutral detergent (20.44 vs. 26.76%) and acid detergent (8.17 vs. 12.66%) fiber. These findings agree with those of Randall and Drew [23]. In addition, fractionated wheat DDGS were higher in lysine (0.88 vs. 0.81%), threonine (1.18 vs. 0.99%), tryptophan (0.39 vs. 0.33%) and the sulfur containing amino acids (1.43 vs. 1.16%). In comparison with soybean meal, fractionated wheat DDGS had a similar crude protein content (45.79 vs. 45.53%) but higher neutral detergent (20.44 vs. 12.19%) and acid detergent (8.17 vs. 5.28%) fiber. In comparison with soybean meal, fractionated wheat DDGS were substantially lower in lysine (0.88 vs. 2.92%), threonine (1.18 vs. 1.75%), and tryptophan (0.39 vs. 0.66%). However, the content of the sulfur containing amino acids (1.43 vs. 1.31%) was higher in fractionated wheat DDGS than soybean meal.

The chemical analyses conducted on the broiler rations confirmed that the diets met the specifications called for in the diet formulation (Table 3). All diets contained approximately the same crude protein content. As the level of fractionated wheat DDGS in the diet increased, the neutral detergent fibre content of the diet decreased reflecting the lower level of this fraction in the fractionated wheat DDGS compared with regular wheat DDGS. All of the experimental diets (Table 3) met or exceeded the amino acid requirements for broilers up to three weeks of age [13]. However, it should not be forgotten that substantial quantities of crystalline lysine had to be added to all diets in order to provide a balanced level of amino acids.

**Table 3:** **Chemical and amino acid analysis of diets fed to determine the effects of regular and fractionated wheat distiller’s dried grains with solubles (DDGS) on the performance of broiler chicks (0–21 d)**^**1**^

	**Level of fractionated wheat DDGS, %**				
	**0**	**5**	**10**	**15**	**20**
Chemical composition, % as fed					
Moisture	8.30	8.34	8.28	8.56	8.45
Ash	6.47	7.13	6.67	6.04	6.27
Crude protein	24.63	24.37	25.10	24.88	25.33
Ether extract	9.35	8.38	9.13	8.03	8.37
Neutral detergent fibre	12.39	12.30	12.18	11.52	11.35
Calcium	1.03	1.01	0.97	1.01	0.98
Total phosphorus	0.72	0.70	0.69	0.73	0.71
Amino acid, % as fed					
Arginine	1.24	1.34	1.25	1.24	1.25
Histidine	0.58	0.61	0.58	0.59	0.60
Isoleucine	0.84	0.91	0.86	0.88	0.91
Leucine	1.51	1.61	1.53	1.56	1.59
Lysine	1.12	1.16	1.21	1.13	1.19
Methionine + cystine	0.92	0.98	0.93	0.90	0.94
Phenylalanine	0.87	0.93	0.88	0.90	0.91
Threonine	0.86	0.92	0.87	0.87	0.89
Tryptophan	0.27	0.27	0.27	0.26	0.28
Valine	1.05	1.13	1.08	1.11	1.13

^1^All data are the results of a chemical analysis conducted in duplicate.

### Apparent total tract digestibility

The effects of graded levels of fractionated wheat DDGS on the ATTD of dry matter and gross energy as well as nitrogen retention are shown in Table 4. The ATTD of dry matter and gross energy were linearly increased (*P* < 0.01) as the level of fractionated wheat DDGS in the diet increased. Nitrogen retention was unaffected by level of fractionated wheat DDGS.

**Table 4:** Dry matter and energy digestibility and nitrogen retention of diets fed to 21 day old broiler chickens containing graded levels of fractionated wheat distiller’s dried grains with solubles (DDGS)

	**Level of fractionated wheat DDGS, %**						***P *Values**		
**Item**	**0**	**5**	**10**	**15**	**20**	**SEM**^**1**^	**Linear**	**Quadratic**	**Cubic**
Dry matter, %	66.3	68.4	69.3	71.0	70.7	0.93	<0.01	0.32	0.78
Energy, %	71.6	73.7	74.4	75.9	75.9	0.85	<0.01	0.36	0.98
Nitrogen retention, %	59.0	61.1	62.8	63.1	62.3	1.43	0.11	0.25	0.87

^1^Standard error of the mean.

Typically, when improvements in nutrient digestibility are observed, they are usually associated with a decrease in the fiber content of the diet. This has been attributed to the fact that most of the fiber is not digested by poultry [24-26]. In addition, dietary fiber reduces nutrient digestibility due to its physiochemical properties, leading to nutrient dilution and a more rapid rate of passage which limits the amount of time available for nutrient breakdown [27]. Therefore, the improvements in dry matter and gross energy digestibility can most likely be attributed to the decrease in fiber content as a result of the post-production fractionation of wheat DDGS.

### Broiler performance

The effects of graded levels of fractionated wheat DDGS on broiler performance are shown in Table 5. Weight gain increased linearly (*P* = 0.05) as the level of fractionated wheat DDGS in the diet increased. Feed intake, feed conversion and mortality were unaffected by the level of fractionated wheat DDGS in the diet (*P* > 0.05). The increased weight gain of broilers fed fractionated wheat DDGS compared with regular wheat DDGS can be attributed to the improvements in dry matter and gross energy digestibility observed as the level of fractionated wheat DDGS in the diet increased.

**Table 5:** Performance of broiler chickens (0–21 d) fed graded levels of fractionated wheat distiller’s dried grains with solubles (DDGS)

	**Level of fractionated wheat distiller’s grains with solubles, (%)**						***P *Values**		
**Item**	**0**	**5**	**10**	**15**	**20**	**SEM**^**1**^	**Linear**	**Quadratic**	**Cubic**
Weight gain, g	966	1,031	1,021	1,026	1,046	23.3	0.05	0.39	0.25
Feed intake, g	1378	1,464	1,430	1,471	1,463	42.8	0.20	0.48	0.61
Feed conversion	1.43	1.42	1.40	1.43	1.40	0.02	0.48	0.88	0.38
Mortality, %	0.00	6.67	0.00	3.33	0.00	1.92	0.29	0.36	0.04

^1^Standard error of the mean.

## Conclusions

Post-production fractionation of wheat DDGS improves its nutritional value by lowering its fiber content and increasing its gross energy and crude protein content. These changes in chemical composition supported increased weight gain of broilers fed wheat DDGS. The remaining fiber rich fraction could be used in diets fed to ruminants.

## Competing interests

The authors declare they have no competing interests.

## Authors’ contributions

PAT participated in the experimental design, data collection, analysis of the data and manuscript preparation. AD supervised the broiler performance trial and edited the manuscript. EB produced the fractionated wheat DDGS and edited the manuscript. All authors read and approved the final manuscript.
